# Pulsatile operation of a continuous-flow right ventricular assist device (RVAD) to improve vascular pulsatility

**DOI:** 10.1371/journal.pone.0195975

**Published:** 2018-04-20

**Authors:** Boon C. Ng, Matthias Kleinheyer, Peter A. Smith, Daniel Timms, William E. Cohn, Einly Lim

**Affiliations:** 1 Department of Biomedical Engineering, University of Malaya, Kuala Lumpur, Malaysia; 2 Texas Heart Institute, Houston, Texas, United States of America; 3 BiVACOR, Inc, Houston, Texas, United States of America; Scuola Superiore Sant'Anna, ITALY

## Abstract

Despite the widespread acceptance of rotary blood pump (RBP) in clinical use over the past decades, the diminished flow pulsatility generated by a fixed speed RBP has been regarded as a potential factor that may lead to adverse events such as vasculature stiffening and hemorrhagic strokes. In this study, we investigate the feasibility of generating physiological pulse pressure in the pulmonary circulation by modulating the speed of a right ventricular assist device (RVAD) in a mock circulation loop. A rectangular pulse profile with predetermined pulse width has been implemented as the pump speed pattern with two different phase shifts (0% and 50%) with respect to the ventricular contraction. In addition, the performance of the speed modulation strategy has been assessed under different cardiovascular states, including variation in ventricular contractility and pulmonary arterial compliance. Our results indicated that the proposed pulse profile with optimised parameters (*A*_*pulse*_ = 10000 rpm and *ω*_*min*_ = 3000 rpm) was able to generate pulmonary arterial pulse pressure within the physiological range (9–15 mmHg) while avoiding undesirable pump backflow under both co- and counter-pulsation modes. As compared to co-pulsation, stroke work was reduced by over 44% under counter-pulsation, suggesting that mechanical workload of the right ventricle can be efficiently mitigated through counter-pulsing the pump speed. Furthermore, our results showed that improved ventricular contractility could potentially lead to higher risk of ventricular suction and pump backflow, while stiffening of the pulmonary artery resulted in increased pulse pressure. In conclusion, the proposed speed modulation strategy produces pulsatile hemodynamics, which is more physiologic than continuous blood flow. The findings also provide valuable insight into the interaction between RVAD speed modulation and the pulmonary circulation under various cardiovascular states.

## Introduction

Left ventricular assist devices (LVADs) have been successfully used for the treatment of end-stage heart failure. However, LVAD recipients still experience significant post-operative complications, such as right heart failure, that occurs in approximately 15% to 30% of patients after LVAD implantation [1, 2]. It has been reported that 42.3% of patients implanted with temporary centrifugal RVADs recovered from right ventricular failure eventually and had their RVAD removed after five days of support [3].

Even though clinical experiences in patients supported with continuous flow rotary blood pump (RBP) have been promising over the past decades, the long term adverse effects of RBP support with attenuated pulsatility on the global and microvascular pulmonary circulation [4–6] remain a long standing controversial subject. The pulsatile nature of the pulmonary blood flow is important for shear stress-mediated release of the endothelium derived nitric oxide (NO) [7], which is in part responsible for the resting pulmonary vasorelaxation and the normalisation of the basal pulmonary resistance [8]. In a nonpulsatile pulmonary circulation, the endothelial NO synthase was depressed, thus leading to a reduction in the endothelial dependent vasorelaxation response of the pulmonary arteries and a subsequent unilateral elevation of the pulmonary arterial pressure (PAP) [9]. On the other hand, several studies have also demonstrated that high pulsatility can significantly upregulate inflammation and cell proliferation in the distal pulmonary microvascular endothelial cells [10, 11], thus suggesting that it is essential to maintain pulse pressure within the physiologic range.

Pump speed modulation has been proposed for the rotary LVAD [12–16] to enhance pulsatility in the systemic circulation. Pirbodaghi et al. [12] made an attempt to synchronize the speed profiles to the natural cardiac cycle by using the R wave detection method. It was concluded that a synchronized pulsing RBP offers a powerful control modality for heart unloading, which plays an essential role in stimulating myocardial recovery and device weaning [17]. Despite the potential benefit of RVAD speed modulation on the pulmonary circulation, most previous investigations on the pulsatile-flow RVAD still relied on the use of conventional volume displacement blood pumps [18, 19] that are larger in size and more susceptible to wear compared to an implantable RBP.

Although interaction between the cardiovascular system and a speed modulated RBP has been explored through numerous in vivo studies [12, 20, 21], such investigations are regarded as inconclusive largely due to the use of healthy animal models, which can poorly represent the actual heart failure scenario. In addition, the reproducibility and controllability of the experimental scenario becomes a major concern when the variation of other cardiovascular states, such as ventricular contraction and arterial compliance, come into play [22]. On the other hand, in vitro and in silico experiments have the advantages of investigating the effect of individual cardiovascular state through parameter adjustment of the mock loop or numerical model. To date, most studies using mock loop [14, 16, 23] and numerical models [13, 14] focused mainly on the configuration of different pump speed/flow profiles. Limited attention has been directed to the implication of variations in the patient conditions, such as improved/deteriorated ventricular contractility [24, 25] and hypertension [26–28]. Therefore, it is worthwhile to investigate the influence of individual cardiovascular state on the resultant pulse pressure, backflow and pump flow produced by the speed modulated RBP.

The current study aims to generate physiological pulse pressure in the pulmonary circulation by modulating the speed of an RVAD in a mock circulation loop. In addition, the effect of the speed modulation strategy is assessed under various cardiovascular states, including variations in right ventricular contractility and pulmonary arterial compliance. A rectangular pulse profile with a predefined pulse width is introduced as the pump speed pattern, with two different timings with respect to the start of ventricular contraction (co- and counter-pulsation). The effect of pump speed modulation on the pulmonary circulation and right ventricular unloading is investigated.

## Materials and methods

### Experimental setup

**Fig 1:**
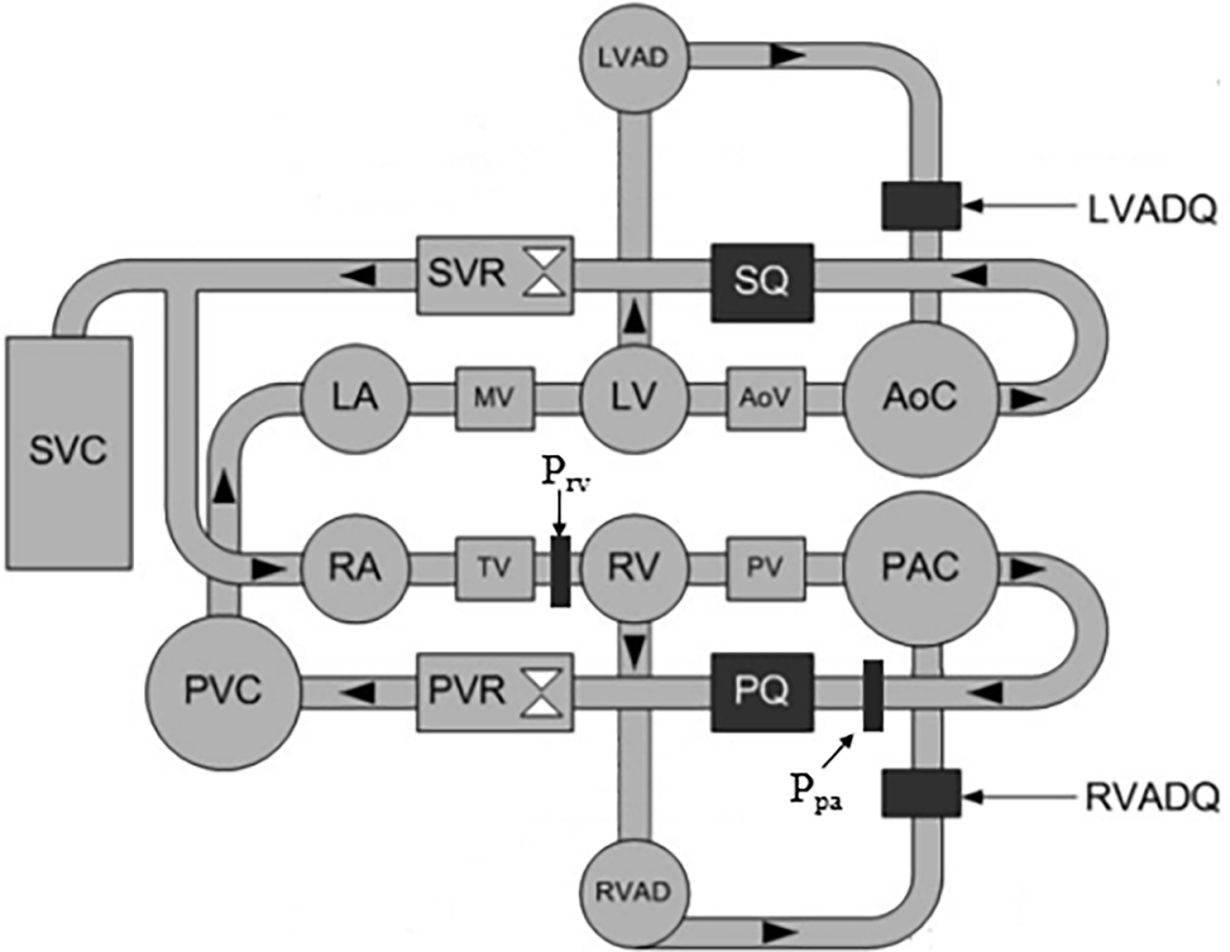
Schematic of the MCL setup with biventricular support. LA, left atrium; MV, mitral valve; LV, left ventricle; AoV, aortic valve; AoC, aortic compliance chamber; SQ, systemic flow meter; SVR, systemic vascular resistance valve; SVC, systemic venous compliance chamber; RA, right atrium; TV, tricuspid valve; RV, right ventricle; PV, pulmonary valve; PAC, pulmonary arterial compliance chamber; PQ, pulmonary flow meter; PVR, pulmonary vascular resistance valve; PVC, pulmonary venous compliance chamber; LVAD, left ventricular assist device; LVADQ, left ventricular assist device flowmeter; RVAD, right ventricular assist device; RVADQ, right ventricular assist device flowmeter; P_pa_, pulmonary arterial pressure sensor; P_rv_, right ventricular pressure sensor.

As depicted in Fig 1, a physical mock circulation loop (MCL) comprising both the systemic and pulmonary circulations was used for this investigation. Briefly, four independent Windkessel chambers were employed to recreate lumped arterial and venous compliances. The compliance of each chamber could be independently varied by changing the volume of air sealed in a given chamber. This was done by adjusting the vertical position of a standard pipe test plug within the pipe chambers. The resistance in the pulmonary and systemic circulatory vasculatures was respectively regulated by changing the input voltage of an electropneumatic pressure regulator, which proportionally occludes the pipe. Both sides of the mock heart consisted of passive atrial and pneumatically actuated ventricular chambers. The atrial and ventricular chambers were represented by clear, vertical polyvinyl chloride (PVC) pipes with tee section connecting the inflow, outflow and the bottom end of the heart chambers. The atrial chambers were open to the atmosphere at the top, which allowed the fluid volume to change in response to the venous return. The ventricular chambers were similar in construction to the atrial chambers, with the addition of an end cap at the top that was tapped with a hose tailpiece to allow compressed air to be input during systole and vented during diastole. Mechanical check valves were used to simulate the mitral, aortic, tricuspid and pulmonary valves to ensure unidirectional flow throughout the circuit. The ventricular systole was controlled through electropneumatic regulators and 3/2-way solenoid valves to provide varying degrees of ventricular contractility, heart rate and systolic duration. A Starling response, which actively regulates the degree of contractility based on the ventricular end diastolic volume, was implemented in both the left and right ventricles. The working fluid was a mixture of water and glycerol (60/40% by mass) that gave a viscosity (3.6 mPa/s at room temperature) within the normal range (3.5–4.5 mPa/s) of the blood viscosity [29]. Systemic and pulmonary flow rates were recorded using magnetic flow meters (IFC010, KROHNE, Duisburg, Germany), while the LVAD and RVAD outlet flow rates were recorded with a clamp-on ultrasonic flow meter (BioProTT^TM^, em-tec, Lerchenberg, Germany). Right ventricular and pulmonary arterial pressures were recorded using silicon-based transducers (PX181B-015C5V, Omega Engineering, Stamford, CT, USA), while right ventricular volume was recorded using a magnetostrictive level sensor (IK1A, GEFRAN, Italy). Detailed documentation of this test rig can be found in [30, 31].

Two LVADs (Thoratec, Heartmate II) were connected to the MCL in a biventricular assist device (BiVAD) configuration. The inflow cannulation sites were the ventricles, while the outflow cannulation sites were the aorta (left pump) and pulmonary artery (right pump). Both pumps were operated using a customized control box which enabled a greater range of set speeds (2000–15000 rpm) compared to the standard Heartmate II controller (6000–15000 rpm). In this study, the MCL was configured to represent a medically treated, severe biventricular heart failure condition with BiVAD support. The slope of the LV and RV end systolic pressure volume relationship (ESPVR) was set at 0.46 mmHg/mL [31] and 0.1 mmHg/mL, respectively, to represent severe left and right heart failure conditions. The resting systemic and pulmonary vascular resistances were set at 1250 dyne s cm^-5^ and 125 dyne s cm^-5^ respectively [32].

### VAD speed modulation

The VADs were first operated at a continuous flow mode, where LVAD and RVAD were regulated at 9300 rpm and 6000 rpm respectively. The pump speeds were determined such that the resultant hemodynamics (left atrial pressure: 11.2 mmHg, right atrial pressure: 8.2 mmHg, aortic pressure: 82.0 mmHg, pulmonary arterial pressure: 18.5 mmHg, blood flow: 5 L/min) was restored back to the normal range of a healthy human.

**Fig 2:**
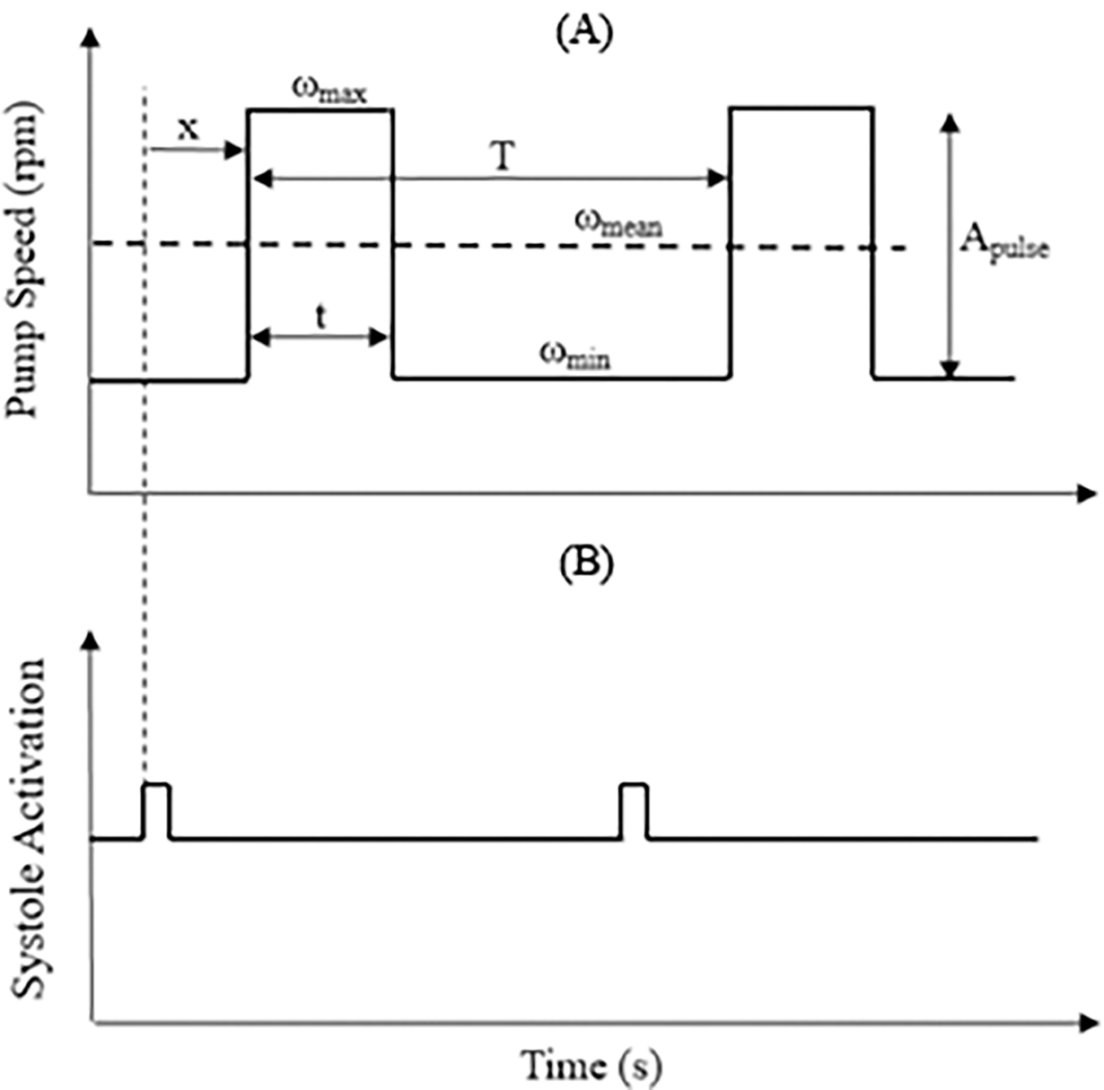
**Synchronisation of the (A) pump speed profile with ECG signal, represented by (B) the rectangular pulse in the MCL.** ω_max_, high pump speed; ω_min_, low pump speed; ω_mean_, the average speed over one cycle; *T*, wave period; *t/T*, pulse width; *A*_*pulse*,_ pulse amplitude; rpm, rotation per minute.

Since the major focus of this study was to investigate the effect of pulsatile flow on the pulmonary circulation, the operation of the left pump remained in the continuous flow mode throughout the study. On the other hand, the right pump speed was modulated based on a rectangular pulse wave shown in Fig 2A to generate pulsatile flow in the pulmonary circulation. The speed profile was characterized by the high pump speed ω_max_, low pump speed ω_min_, wave period *T*, pulse width *t/T*, pulse amplitude *A*_*pulse*_ as well as the average speed over one cycle ω_mean_. In practice, synchronisation of the pulse waveform with the heartbeat can be realized through detection of the QRS complexes in the ECG signal [12]. During the MCL operation, the rectangular pulse shown in Fig 2B was generated in each cycle, replicating the start of the systole in the ventricular chamber. This rectangular pulse was used as the reference to set the phase shift *x* between the native cardiac cycle and the pump pulses.

The mean pump speed was computed by averaging the area covered by the modulated speed profile in each cycle over the entire cycle period.

$$\omega_{mean}=A_{pulse}.\frac{t}{T}+\omega_{min}$$ 1)

In this study, the period *T* and pulse width *t/T* of the speed waveform were fixed at 1 s and 0.3 respectively, determined based on the predefined heartbeat (60 bpm) and systolic time fraction (30%) for the MCL. By substituting the values of the known variables in Eq (1) and deriving it against *A*_*pulse*_, a case specific correlation between *A*_*pulse*_ and *ω*_*min*_ was obtained as follow:

$$A_{pulse}=\frac{6000-\omega_{min}}{0.3}$$ 2)

In this study, three combinations of *A*_*pulse*_ and *ω*_*min*_, which produced the same average pump revolution per each heartbeat (i.e. 6000 rpm as determined earlier to restore the normal hemodynamic), were evaluated, as shown in Table 1. In addition, the effect of co- and counter-pulsation was investigated by varying the phase shift *x* between the native cardiac cycle and the pump pulses in the MCL.

**Table 1:** Variation of pulsing parameters, speed waveforms, phase shift, ventricular contractility and pulmonary arterial compliance.

Variation	
*A*_*pulse*_ and *ω*_*min*_ (rpm)	[13500 2000]; [10000 3000]; [6700 4000]
Phase shift x	0 s (co-pulsation) and 0.5 s (counter-pulsation)
Right and left ventricular contractilities	[Severe Severe], [Mild Severe]
Pulmonary arterial compliance (mL/mmHg)	2.5, 3.2 (baseline), 4.8

*A*_*pulse*_: pulse amplitude; ω_min_: minimum pump speed.

### Variations in the cardiovascular states

To investigate the effect of right ventricular recovery, the ESPVR slope for the right ventricle was increased from 0.1 mmHg/mL to 0.23 mmHg/mL. In addition, the effect of pulmonary arterial compliance was investigated by evaluating three different pulmonary arterial compliance settings using the MCL: 2.5, 3.2 and 4.8 mL/mmHg (Table 1). It is noteworthy to highlight that even though the clinically recorded pulmonary arterial compliance values amongst patients with hypertension were generally lower than those without hypertension, the measurements obtained for both categories were widely distributed and densely overlapped [33]. Therefore, in this study, the setting of 3.2 mL/mmHg was chosen to represent the average pulmonary arterial compliance for patients without hypertension. For patients with hypertension, an average pulmonary arterial compliance of 2.5 mL/mmHg was selected. On the other hand, the setting of 4.8 mL/mmHg used in this study to represent high pulmonary arterial compliance was slightly above average amongst patients without hypertension [33, 34].

### Data analysis

In this study, analysis of the hemodynamic parameters were performed in MATLAB (MathWorks, Natick, MA) based on the in-vitro measurements collected from the mock circulatory loop. The pulsatility in the pulmonary circulation (indicated by pulse pressure) was characterized by the difference between the maximum pulmonary arterial systolic pressure and minimum pulmonary arterial diastolic pressure. The mean pressure and flow were quantified based on the average value of the measured pressures and flows over 20 cardiac cycles. Stroke work was defined as the encompassed area within a right ventricular pressure-volume (PV) loop, which also corresponds to the energy imparted by the right ventricle to drive blood. The stroke volume was obtained by subtracting the end-systolic volume from the end-diastolic volume of the right ventricle. The risk of backflow through RVAD was quantified based on the minimum value of the pump flow. Negative measurement of the pump flow indicates the event of backward flow across the pump.

## Results

### Effect of VAD speed modulation

**Fig 3:**
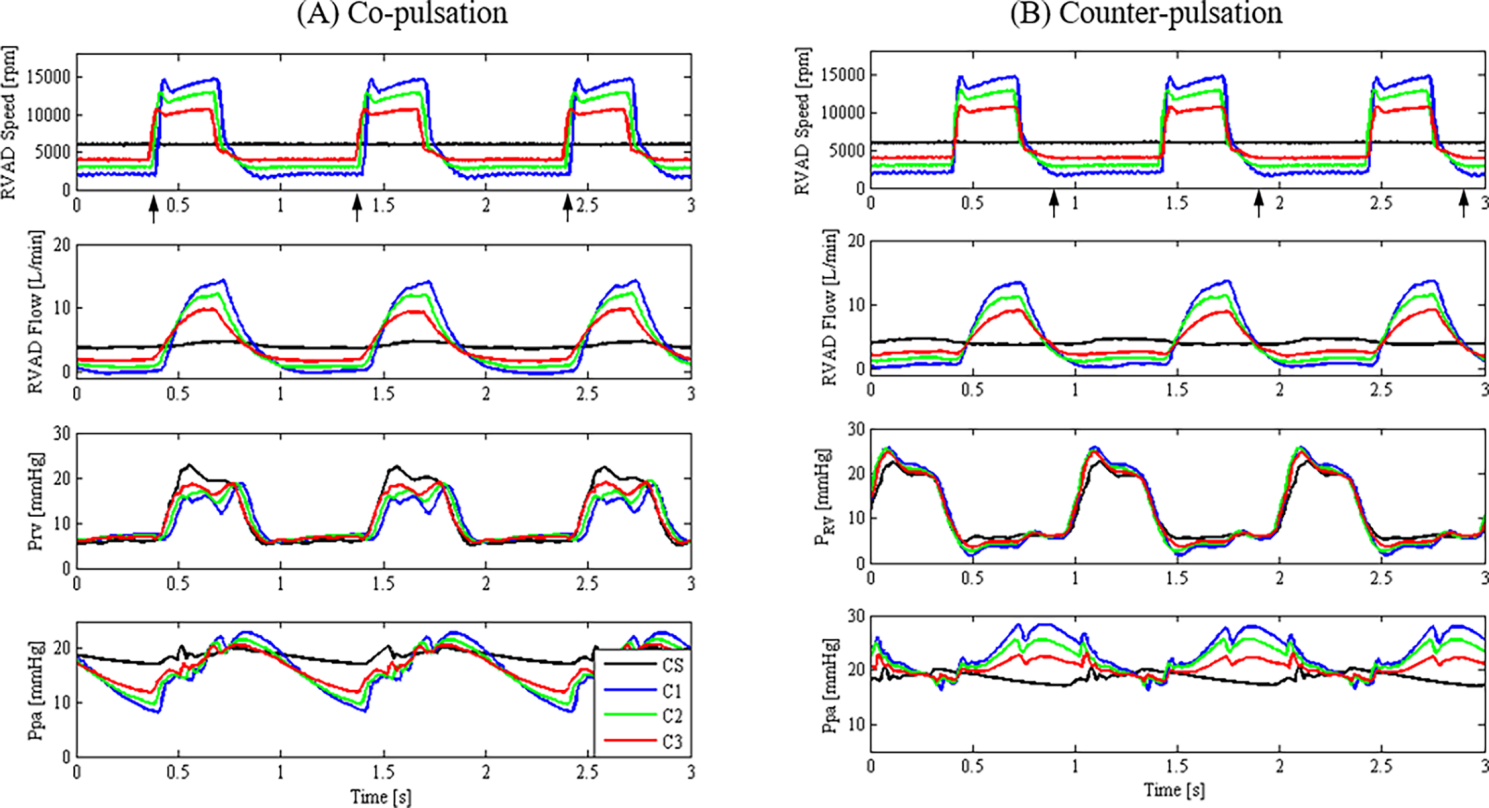
The changes in RVAD flow, right ventricular pressure and pulmonary arterial pressure with different pump speed configurations for co- and counter-pulsation modes. CS: constant speed; C1: *A*_*pulse*_ = 13500 rpm and *ω*_*min*_ = 2000 rpm; C2: *A*_*pulse*_ = 10000 rpm and *ω*_*min*_ = 3000 rpm; C3: *A*_*pulse*_ = 6700 rpm and *ω*_*min*_ = 4000 rpm; RVAD: right ventricular assist device; P_RV_, right ventricular pressure; Ppa, Pulmonary arterial pressure; black arrow: the beginning of systole.

Fig 3 illustrates the effects of varying pulsing profiles and phase shifts on the hemodynamics. As compared to the constant speed mode, higher pulse pressure was achieved through pump speed modulation. The increment in pulse pressure appeared to be more remarkable through co-pulsation, resulting from a larger fluctuation in pump flow throughout the cardiac cycle (Fig 3 & Table 2). Higher increment in maximum pump flow during systole through co-pulsation was attributed to the synergetic effects between ventricular contraction and elevated pump speed, while the drastic decrement in minimum pump flow during diastole was seen as the outcome of a reduction in pump speed and ventricular relaxation. On the contrary, the increment in pulse pressure produced by counter-pulsation was lesser as compared to co-pulsation due to the competing effects between the reduced pump speed and ventricular contraction during systole as well as the elevated pump speed and reduced ventricular pressure during diastole.

**Table 2:** Effect of varying pump speed configurations on the hemodynamics.

	CS	C1	C2	C3
**Co-pulsation**				
SW_RV_ (J)	0.082	0.089	0.093	0.095
PP_pa_ (mmHg)	3.6	13.3	11.0	8.7
${\bar{P}}_{\mathrm{pa}}$ (mmHg)	19.3	17.0	17.1	17.5
${\bar{Q}}_{t}$ (L/min)	5.1	5.0	5.0	5.1
${\bar{Q}}_{\mathrm{RVAD}}$ (L/min)	4.4	4.8	4.6	4.5
*Q*_RVAD,max_ (L/min)	-	14.3	12.1	9.8
*Q*_RVAD,min_ (L/min)	-	-0.4	0.3	1.2
**Counter-pulsation**				
SW_RV_ (J)	0.082	0.032	0.048	0.053
PP_pa_ (mmHg)	3.6	11.8	9.6	6.3
${\bar{P}}_{\mathrm{pa}}$ (mmHg)	19.3	23.6	22.6	21.6
${\bar{Q}}_{t}$ (L/min)	5.1	5.4	5.3	5.2
${\bar{Q}}_{\mathrm{RVAD}}$ (L/min)	4.4	4.6	4.5	4.3
*Q*_RVAD,max_ (L/min)	-	13.3	11.4	8.8
*Q*_RVAD,min_ (L/min)	-	-0.1	0.5	1.4

P: Pressure; PP: Pulse pressure; $\bar{Q}$; average flow; RV: right ventricle; SW: Stroke work; min: minimum; pa: pulmonary artery; t total. (CS: constant speed; C1: *A*_*pulse*_ = 13500 rpm and *ω*_*min*_ = 2000 rpm; C2: *A*_*pulse*_ = 10000 rpm and *ω*_*min*_ = 3000 rpm; C3: *A*_*pulse*_ = 6700 rpm and *ω*_*min*_ = 4000 rpm)

As compared to co-pulsation, higher total blood flow (pump flow + flow across the pulmonary valve) in the pulmonary circulation was produced through counter-pulsation. This was in large part attributed to the higher pulmonary valve flow (Table 2) in counter-pulsation, which produced the minimum speed during systole. Higher flow in the pulmonary circulation has thereby led to an increase in the mean pulmonary arterial pressure.

**Fig 4:**
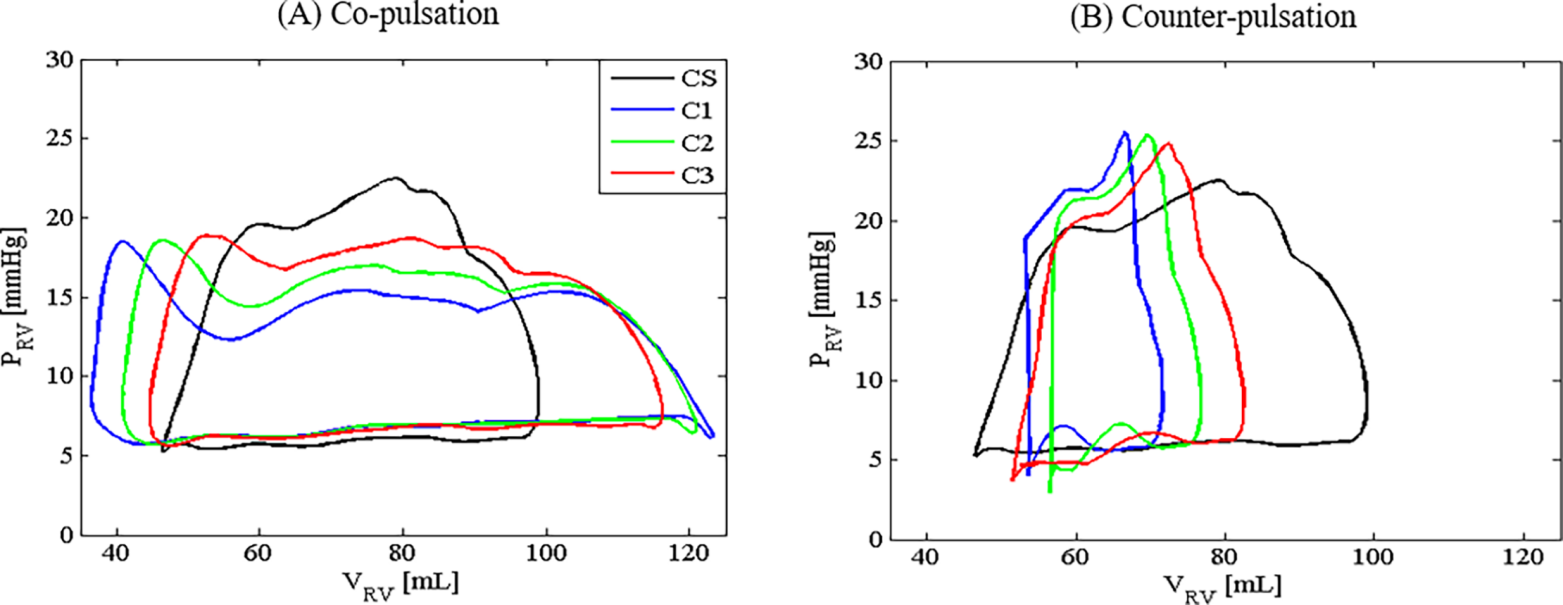
The PV loops produced through speed modulation with co- and counter-pulsation. CS: constant speed; C1: *A*_*pulse*_ = 13500 rpm and *ω*_*min*_ = 2000 rpm; C2: *A*_*pulse*_ = 10000 rpm and *ω*_*min*_ = 3000 rpm; C3: *A*_*pulse*_ = 6700 rpm and *ω*_*min*_ = 4000 rpm; P_RV_, right ventricular pressure; V_RV_, right ventricular volume.

As shown in Fig 4A, speed modulation with co-pulsation delivered significantly higher end diastolic volume with a minor reduction in the end systolic volume, thus resulting in a higher stroke work as compared to that produced by the constant speed mode (Table 2). On the contrary, counter-pulsation produced the highest pump speed as well as pump flow during diastole, thereby resulting in a lower end diastolic volume and a weaker ventricular contraction during systole. As a result, the PV loop (Fig 4B) as well as the corresponding stroke work (Table 2) produced during counter-pulsation was smaller as compared to the constant speed mode. Among the three speed modes (constant speed, co-pulsation and counter-pulsation), lowest stroke work was generated under counter-pulsation, suggesting that mechanical workload of the right ventricle can be efficiently mitigated via counter-pulsing the pump speed.

As compared to other pump speed configurations (i.e. CS, C2 and C3), C1 generated the highest pulmonary arterial pulse pressure due to its highest speed amplitude *A*_*pulse*_ and lowest minimum speed *ω*_*min*_ (Table 2). The pulse pressures produced by C1 and C2 under both pulsing modes were found to be within the normal range (9–15 mmHg) of a healthy human [35, 36]. Nevertheless, as shown in Table 2, the major drawback of having a speed profile with high *A*_*pulse*_ and low *ω*_*min*_ (as in the case of C1) is the potential backflow through the pump during diastole (minimum pump flow *Q*_RVAD,min_ = -0.4L/min, indicating a backward flow of 0.4 L/min across the pump). The undesirable backflow could be addressed by setting a higher value for the minimum pump speed *ω*_*min*_. For an instance, it can be observed from Table 2 that when *ω*_*min*_ was increased to 3000 rpm (as in the case of C2), no backflow event was observed throughout the whole pulsing cycle. Alternatively, the risk of backflow occurrence could also be significantly reduced by switching the speed modulation to the counter-pulsation mode. As indicated in Table 2 for case C1, the backflow generated under the co-pulsation mode was reduced when the pump was operated under the counter-pulsation mode. The reduced backflow risk is attributed to the fact that speed modulation under counter-pulsation delivered a higher upstream pressure (P_RV_) and thereby a lower pressure gradient across the pump by setting the pump speed to *ω*_*min*_ during systole.

### Effect of right ventricular contractility

**Fig 5:**
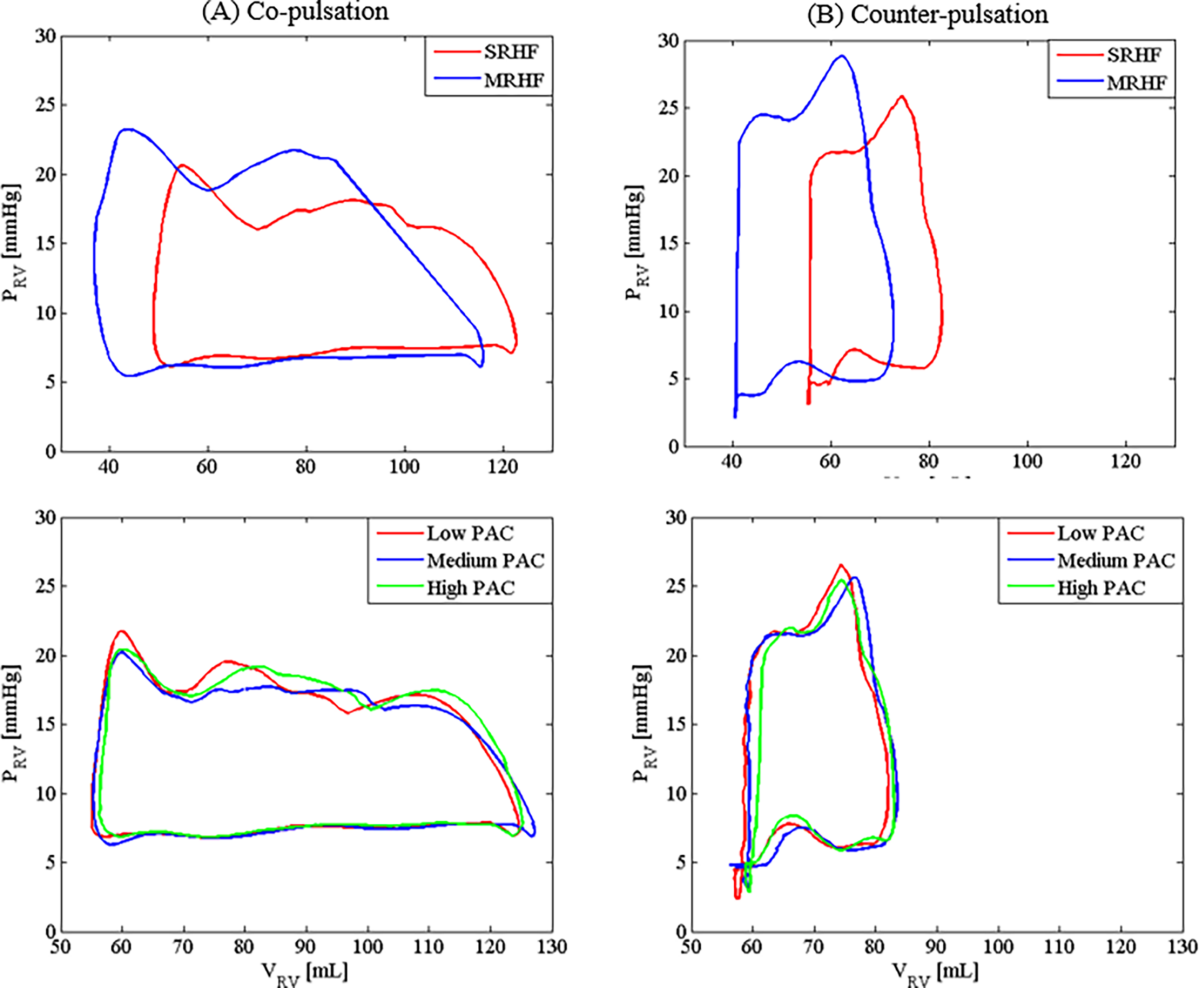
Right ventricular PV loops for the mild and severe right heart failure (MRHF and SRHF) scenarios as well as at different pulmonary arterial compliance (PAC) levels. MRHF, mild right heart failure (ESPVR slope = 0.23 mmHg/mL); SRHF, severe right heart failure (ESPVR slope = 0.1 mmHg/mL); P_RV_, right ventricular pressure; V_RV_, right ventricular volume; PAC, pulmonary arterial compliance.

In comparison to the baseline condition, an improved right heart failure condition led to a larger and leftward shift of the PV loop (Fig 5). Elevated ventricular stroke work in response to an improved right heart contractility resulted in an increase in the total flow ${\bar{Q}}_{t}$ and the mean pulmonary arterial pressure (Table 3). Attributing to the higher pump pressure gradient (higher *P*_*pa*_ and lower *P*_*rv*_) during the diastolic phase, a lower minimum pump flow can be observed in the scenario of mild right heart failure, which eventually led to a higher backflow risk. An improvement in the contractility has also resulted in a reduction in the diastolic pressure and volume (see PV loop in Fig 5), particularly with counter-pulsation, which indicates a higher suction risk in the right ventricle. In addition, higher stroke volume (Table 3 & Fig 5) can be observed with an improvement in the right heart contractility due to a greater contribution of flow by the native heart (i.e. flow across the pulmonary valve).

**Table 3:** Effect of varying right ventricular contractility on the hemodynamics during speed modulation using pulsing profile C2.

	Severe RHF	Mild RHF
**Co-pulsation**		
SW_RV_ (J)	0.093	0.15
PP_pa_ (mmHg)	11.0	12.7
${\bar{P}}_{\mathrm{pa}}$ (mmHg)	17.1	19.9
SV(mL)	73	80
${\bar{Q}}_{t}$ (L/min)	5.0	5.2
${\bar{Q}}_{\mathrm{RVAD}}$ (L/min)	4.6	4.4
*Q*_RVAD,min_ (L/min)	0.3	-0.1
**Counter-pulsation**		
SW_RV_ (J)	0.048	0.082
PP_pa_ (mmHg)	9.6	9.4
${\bar{P}}_{\mathrm{pa}}$ (mmHg)	22.6	26.3
SV (mL)	27	32
${\bar{Q}}_{t}$ (L/min)	5.3	5.5
${\bar{Q}}_{\mathrm{RVAD}}$ (L/min)	4.5	4.3
*Q*_RVAD,min_ (L/min)	0.5	0.2

P: Pressure; PP: Pulse pressure; $\bar{Q}$; average flow; RHF: right heart failure; RV: right ventricle; SV:Stroke volume; SW: Stroke work; min: minimum; pa: pulmonary artery; t: total.

### Effect of pulmonary arterial compliance

The average total flow and pulmonary arterial pressure remained nearly unchanged in response to variations in the pulmonary arterial compliance (Table 4). Pulse pressure varied significantly, as *P*_*pa*_ oscillated more swiftly with larger amplitude when the pulmonary arterial compliance was reduced. This is in agreement with the in vivo findings in [37] that reported an immediate increase in the pulmonary arterial pulse pressure with stiffening of the main pulmonary artery. On the other hand, the PV loops produced by the MCL in all three scenarios were seen to be almost identical.

**Table 4:** Effect of varying pulmonary arterial compliance on the hemodynamics during speed modulation using pulsing profile C2.

	Low PAC (2.5 mL/mmHg)	Medium PAC (baseline, 3.2 mL/mmHg)	High PAC (4.8 mL/mmHg)
**Co-pulsation**			
SW_RV_ (J)	0.089	0.093	0.091
PP_pa_ (mmHg)	14.8	11.0	9.4
${\bar{P}}_{\mathrm{pa}}$ (mmHg)	17.4	17.1	16.9
${\bar{Q}}_{t}$ (L/min)	5.0	5.0	5.0
${\bar{Q}}_{\mathrm{RVAD}}$ (L/min)	4.5	4.6	4.6
*Q*_RVAD,min_ (L/min)	0.2	0.3	0.5
**Counter-pulsation**			
SW_RV_ (J)	0.046	0.048	0.040
PP_pa_ (mmHg)	10.0	9.6	8.2
${\bar{P}}_{\mathrm{pa}}$ (mmHg)	23.0	22.6	22.0
${\bar{Q}}_{t}$ (L/min)	5.2	5.3	5.2
${\bar{Q}}_{\mathrm{RVAD}}$ (L/min)	4.5	4.5	4.6
*Q*_RVAD,min_ (L/min)	0.4	0.5	0.6

P: Pressure; PAC: pulmonary arterial compliance; PP: Pulse pressure; $\bar{Q}$; average flow; RV: right ventricle; SW: Stroke work; min: minimum; pa: pulmonary artery; t: total.

## Discussion

One of the major objectives of this study is to investigate the feasibility of enhancing pulse pressure in the pulmonary artery through modulation of the RVAD speed. Our results (Table 2) demonstrated that proper parameterization of the pulse profile could maintain pulmonary arterial pulse pressure within the physiological range of a healthy human (9–15 mmHg) [35, 36]. Generating a physiological pulse pressure in the pulmonary circulation is essential, as inadequate pulse pressure has been reported as the potential factor leading to an altered endothelial dependent vasorelaxation response and vascular remodelling [38], while excessive pulse pressure may result in a deteriorated pulmonary microcirculation [39].

Optimization of the pulmonary arterial pulse pressure is a multiobjective task as it could come at the expense of several adverse events such as pump backflow, ventricular suction as well as an increase in the ventricular stroke work. To date, studies related to RBP speed modulation [12, 13, 20, 21] have mainly focused on optimizing the pulse pressure as well as reducing the ventricular stroke work, without considering the negative consequences such as pump back flow and suction. In view of a typical Differential Pressure vs Flow Curve (H-Q curve) for a RBP, a blood pump is susceptible to negative flow when it is subjected to higher pump head while operating at a low pump speed [40]. Since commercially available LVADs are frequently used as RVADs in the patients, the device has to be operated at a much lower speed setting to accommodate for the remarkably lower pulmonary vascular resistance. As illustrated in our results (Table 2 & Fig 3), operating the VAD at a low pump speed during diastole may result in backflow (particularly in the case of co-pulsation) due to the large pressure difference between the right ventricle and the pulmonary artery. In view of the adverse events brought by pump backflow, which include an increase in device-related hemolysis and right ventricular end-diastolic volume [41], it is necessary to take this into account while optimizing for pulsatility in the circulation. While our study has indicated that selection of a higher minimum pump speed during diastole can potentially overcome the backflow issue, it comes at the expense of a lower pulsing speed amplitude and therefore a lower pulsatile pressure. Consequently, a trade-off between these two competing criteria should be taken into careful consideration in the selection of the most appropriate pump speed profile.

Patients supported with temporary RVADs post LVAD implantation may benefit from speed modulation of the RVAD with counter-pulsation, as myocardial recovery is favoured with a more pronounced unloading of the right ventricle [3, 42]. This is clearly demonstrated in our results (Table 2 & Fig 4), which showed a major reduction in the stroke work and the ventricular end diastolic volume with the counter-pulsation mode as compared to that produced by the co-pulsation mode. Similar strategy of speed modulation has also been demonstrated by Pirbodaghi, Axiak (12) in unloading the left ventricles in an animal test. Clinically, it has been reported that the implantation of pulsatile LVAD in pediatrics was subsequently followed by an early and mid-term left ventricle unloading, as expressed by a decrease in LV volumes and diameters at echocardiogram [43]. However, there is no further investigation on the possibility of prolonging the ventricular unloading effect by phase shifting the pump pulsation with respect to the native heart pulse. This strategy is particularly useful in the pump weaning process, in which patients require an optimization of the stroke work during cardiac recovery, while maintaining end organ perfusion.

Variations in the cardiovascular states, such as ventricular contractility and pulmonary arterial compliance, significantly influence the pump speed modulation performance (Tables 3 and 4, Fig 5). Since VAD-assisted patients are constantly subjected to these variations, determination of a fixed pump speed pattern for an implantable rotary blood pump that could accommodate for a wide range of clinical scenario is a challenging task. With an improvement in the right heart contractility, stroke volume increased, leading to an increase in the pulse pressure (Table 3, Fig 5) due to a close correlation between these two parameters. As suggested by both clinical studies and theoretical proofs based on the Windkessel model, an elevation in the pulse pressure accounted for a rise in the stroke volume [44, 45]. In the event of high pulsatility due to the synergy effect of pump speed pulsation and improved heart contractility, our pump speed modulation strategy allows for clinical intervention through optimization of speed amplitude and minimum pump speed such that the pulse pressure can be maintained within the physiological range.

Pulmonary hypertension is associated with the narrowing and stiffening of the pulmonary arteries, which are often numerically represented by an increment in the PVR and a decrement in the pulmonary vascular compliance [46]. The correlation between pulse pressure and arterial compliance can be observed in our in-vitro results, where increment in the proximal arterial compliance resulted in a reduction in the pulse pressure magnitude (Table 4). On the other hand, the effect of PVR on the pulse pressure has also been investigated on the MCL but was not reported in this paper, as the variation in pulse pressure with respect to PVR was found to be much less significant. Our results is in line with the findings of previously published finding that PVR mainly affects the steady component of the total RV workload, while the oscillatory component is contributed by the pulmonary vascular compliance [47].

This outcome is in agreement with the clinical use of pulse pressure as an indirect measure of vascular stiffness and pulsatile load [48]. The need to consider the elastic properties of the pulmonary circulation in addition to the PVR is apparent because abnormal pulsatile load may have detrimental effects on the weakened heart and cause adverse ventricular remodelling [27]. Unlike the attenuated pulsatility generated by the CF-RVAD, the time domain analysis of the restored pulse pressure using the speed modulation technique could provide valuable information on the pulsatile arterial load. Since arterial stiffening is a common consequence of biological aging and arteriosclerosis [28, 49], rotary pump speed modulation in patients of older age or with symptom of arterial wall thickening should be regulated with a lower pump speed amplitude, such that pulse pressure remains within the physiological range and cardiovascular events such as stroke can be avoided [50].

In addition to PVR, previous studies have suggested that arterial stiffening in hypertension partially contributes to an increase in the right ventricular afterload [51]. Under normal condition, reflected waves from the pulmonary vasculature return to the pulmonary valve during diastole and therefore do not affect right ventricular ejection. With a reduction in the pulmonary artery compliance, reflected waves reach the pulmonary valve during mid or late systole due to an increase in the pulse wave velocity, thus resulting in a higher right ventricular afterload [47, 52]. Nevertheless, in our MCL study, PV loops produced by different pulmonary arterial compliances were observed to overlap with one another (Fig 5). This observation could be attributed to the lack of wave reflection in our MCL system, which eventually leads to the trivial effect of the pulmonary arterial compliance on the right ventricular afterload.

The present study provided a pump speed profile that can be flexibly parameterized (t/T, T, A_pulse_, ω_min_ and phase shift) in order to reproduce physiological pulsatility in the pulmonary circulation. In this study, the variation in A_pulse_, ω_min_ and phase shift have been investigated, while t/T and T were kept constant. The flexibility in parameter variation paved way for the future development of a physiological controller, which can potentially adapt ω_min_ and A_pulse_ based on the feedback of the pulse pressure in the pulmonary artery and the flow through the pump. The advantage of the proposed speed profile is that the speed amplitude can be arbitrarily varied within the operating range, while maintaining the overall average speed. By adopting this method, the physician has the flexibility to optimize the pump speed amplitude and thereby the pulse pressure without significantly affecting the mean pump flow, given that other cardiovascular states (i.e. PVR, SVR and pulmonary vascular compliance) were held constant. In our result, the average pump flow varied slightly in each scenario despite the setting of a constant average pump speed. This observation is mainly attributed to the incapability of a linear PID with fixed control parameters to optimally cope with a wide range of MCL settings for different scenarios, resulting in the deviation of the actual speed trajectories from the desired profiles. Similar observation can be found in [12], in which a PID based speed controller is used to generate rectangular waveform.

A number of important limitations exist in our study. Clearly, an investigation using MCL is not intended to replace the importance and significance of in vivo models. Specifically, the validated MCL is an idealized model of the systemic and pulmonary circulations and is unable to mimic the neurohumoral responses. The blood circulation was modelled using rigid piping, with vascular compliance represented by the lumped parameter Windkessel compliance elements, while in an actual scenario the compliance is distributed along the venous and the arterial trees. Hence, the MCL was unable to replicate phenomena pertinent to a distributed system, such as the occurrence of wave reflection at several sites along the vessels, including the arterial branches, which may affect pulse pressure. Though incapable of replicating all expected clinical responses, our in vitro study has provided preliminary insights into the interaction between the pulsing RVAD and the cardiovascular system. In addition, in vitro studies serve as an important tool to evaluate the feasibility of new concepts and enable the development of novel control strategies that can be translated into an animal model for viability assessment of clinical application.

The present study involved only RVAD speed pulsing, with the LVAD remained under continuous flow operation. Therefore, another major limitation of the reported findings was that the influence of LVAD speed modulation on the pulmonary circulation was not taken into account. Future studies may investigate the interaction between the left and right hearts that are both supported by speed modulated RBPs. In addition, only limited cardiovascular states have been investigated in this study and the effect of the system parameters has been individually evaluated by varying the value of one single parameter at a time. In an actual clinical scenario, the influence of cardiovascular states on the pulsatility may be more complex as it involves the combined effect of multiple parameters that may vary simultaneously. In future studies, the range of variations in the studied parameters shall be expanded along with the investigation of the effect of speed modulation strategy under varying heart rates, amplitudes and pulse widths of the speed waveform.

## Conclusion

In this study, modulation of RVAD speed using a rectangular pulse profile has been assessed under different cardiovascular states. Our results indicated that proper patient specific optimization of the pump speed profile is able to produce physiological pulse pressure in the pulmonary circulation without causing pump backflow during diastole. Synchronization of the pump speed waveform to the heart beat is essential in optimizing the right ventricular workload. Gradual improvement in the cardiac function may eventually increase the risk of pump backflow and ventricular suction with pump speed modulation, while stiffening of the pulmonary artery leads to an increase in the pulse pressure. Further optimization of the speed modulation parameters can be performed through the implementation of a feedback control scheme that constantly adjusts the speed profile in response to the time varying cardiovascular states.

## References

[1] Kucuker SA, Stetson SJ, Becker KA, Akguel A, Loebe M, Lafuente JA, et al Evidence of Improved Right Ventricular Structure after LVAD support in Patients with End-Stage Cardiomyopathy. J Heart Lung Transpl. 2004;23(1):28–35. 10.1016/s1053-2498(03)00057-3 14734124

[2] Goldstein DJ, Oz MC, Rose EA. Implantable Left Ventricular Assist Devices. The New England Journal of Medicine. 1998;339:1522–33. doi: 10.1056/NEJM199811193392107 9819452 10.1056/NEJM199811193392107

[3] Saito S, Sakaguchi T, Miyagawa S, Nishi H, Yoshikawa Y, Fukushima S, et al Recovery of Right Heart Function with Temporary Right Ventricular Assist Using a Centrifugal Pump in Patients with Severe Biventricular Failure. The Journal of Heart and Lung Transplantation. 2012;31(8):858–64. doi: 10.1016/j.healun.2012.03.002 22609185 10.1016/j.healun.2012.03.002

[4] Furusa A, Brawley RK, Gott VL. Pulsatile Cavopulmonary Artery Shunt: Surgical Technique and Hemodynamic Characteristics. J Thorac Cardiov Sur. 1972;63(3):495–500. 5011291

[5] Rai JU, Kaapa P, Anderson J. Effect of Pulsatile Flow on Microvascular Resistance in Adult Rabbit Lung. Journal of Applied Physiology. 1992;72(1):73–81. doi: 10.1152/jappl.1992.72.1.73 1537745 10.1152/jappl.1992.72.1.73

[6] Taguchi S, Yozu R, Iseki H, Soma Y, Inoue T. Effects of Nonpulsatile and Pulsatile Right Ventricular Bypass on Pulmonary Circulation. Transaction American Society for Artificial Internal Organs. 1988;34(3):213–21. 3058175

[7] Khambadkone S, Li J, de-Leval MR, C S., Deahfield JE, Redington AN. Basal Pulmonary Vascular Resistance and Nitric Oxide Responsiveness Late After Fontan-Type Operation. Circulation. 2003;107(25):3204–8. doi: 10.1161/01.CIR.0000074210.49434.40 12821557 10.1161/01.CIR.0000074210.49434.40

[8] Cooper CJ, Landzberg MJ, Anderson TJ, Charbonneau F, Creager MA, Ganz P, et al Role of Nitric Oxide in the Local Regulation of Pulmonary Vascular Resistance in Humans. Circulation. 1996;93(2):266–71. 8548898 10.1161/01.cir.93.2.266

[9] Henaine R, Vergnat M, Bacha E, Baudet B, Lambert V, Belli E, et al Effects of Lack of Pulsatility on Pulmonary Endothelial Function in the Fontan Circulation. Congenital Heart Disease. 2013;146(3):522–9. 10.1016/j.jtcvs.2012.11.031 23219498

[10] Li M, Scott DE, Shandas R, Stenmark KR, Tan W. High Pulsatility Flow Induces Adhesion Molecule and Cytokine mRNA Expression in Distal Pulmonary Artery Endothelial Cells. Ann Biomed Eng. 2009;37(6):1082–92. doi: 10.1007/s10439-009-9684-3 19340571 10.1007/s10439-009-9684-3 PMC3057109

[11] Tan Y, Tseng P-O, Wang D, Zhang H, Hunter K, Hetzberg J, et al Stiffening-Induced High Pulsatility Flow Activities Endothelial Inflammation via a TLR2/NF-κB Pathway. Plos One. 2014;9(7):1–14. 10.1371/journal.pone.0102195 PMC4100881 25029271

[12] Pirbodaghi T, Axiak S, Weber A, Gempp T, Vandenberghe S. Pulsatile Control of Rotary Blood Pumps: Does the Modulation Waveform Matter? J Thorac Cardiov Sur. 2012;144(4):970–7. 10.1016/j.jtcvs.2012.02.015 22418246

[13] Ising M, Warren S, Sobieski MA, Slaughter MS, Koenig SC, Giridharan GA. Flow Modulation Algorithms for Continuous Flow Left Ventricular Assist Devices to Increase Vascular Pulsatility: A Computer Simulation Study. Cardiovascular Engineering and Technology. 2011;2(2):90–100.

[14] Bearnson GB, Olsen DB, Khanwilkar PS, Long JW, Allaire PE, Maslen EH. Pulsatile Operation of a Centrifugal Ventricular Assist Device with Magnetic Bearings. ASAIO Journal. 1996;42(5):620–4. 10.1097/00002480-199609000-00062 8944955

[15] Qian KX. Pulsatile Impeller Heart: A Viable Alternative to a Problematic Diaphragm Heart. Medical Engineering & Physics. 1996;18:57–66. 8771040 10.1016/1350-4533(95)00010-0

[16] Khalil HA, Kerr DT, Schusterman MA, Cohn WE, Frazier OH, Radovancevic B. Induced Pulsation of a Continuous-Flow Total Artificial Heart in a Mock Circulatory System. The Journal of Heart and Lung Transplantation. 2010;29(5):568–73. doi: 10.1016/j.healun.2009.12.004 20153967 10.1016/j.healun.2009.12.004

[17] Burkhoff D, Klotz S, Mancini D. LVAD-Induced Reverse Remodeling: Basic and Clinical Implications for Myocardial Recovery. Journal of Cardiac Failure. 2006;12(3):227–39. doi: 10.1016/j.cardfail.2005.10.012 16624689 10.1016/j.cardfail.2005.10.012

[18] Karimova A, Pockett CR, Lasuen N, Dedieu N, Rutledge J, Fenton M, et al Right Ventricular Dysfunction in Children Supported with Pulsatile Ventricular Assist Devices. The Journal of Thoracic and Cardiovascular Surgery. 2014;147(5):1691–7. doi: 10.1016/j.jtcvs.2013.11.012 24342898 10.1016/j.jtcvs.2013.11.012

[19] Chen JM, Levin HR, Catanese J, Sistino JJ, Landry DW, Rose EA, et al Use of a Pulsatile Right Ventricular Assist Device and Continuous Arteriovenous Hemodialysis in a 57-Year-Old Man with a Pulsatile Left Ventricular Assist Device. J Heart Lung Transpl. 1995;14(1):186–91. 7727468

[20] Pirbodaghi T, Weber A, Axiak S, Carrel T, Vandenberghe L. Asymmetric Speed Modulation of a Rotary Blood Pump Affects Ventricular Unloading. Eur J Cardio-Thorac. 2012;43(2):383–8. 10.1093/ejcts/ezs299 22689185

[21] Ando M, Nishimura T, Takewa Y, Yamazaki K, Kyo S, Ono M, et al Electrocardiogram-Synchronized Rotational Speed Change Mode in Rotary Pumps Could Improve Pulsatility. Artifical Organs. 2011;35(10):941–7. 10.1111/j.1525-1594.2011.01205.x 21615427

[22] Vollkron M, Schima H, Huber L, Wieselthaler G. Interaction of the Cardiovascular System with an Implanted Rotary Assist Device: Simulation Study with a Refined Computer Model. Artifical Organs. 2002;26(4):349–59. 10.1046/j.1525-1594.2002.06870.x 11952506

[23] Jahren SE, Ochsner G, Shu F, Amacher R, Antaki JF, Vandenberghe L. Analysis of Pressure Head-Flow Loops of Pulsatile Rotodynamic Blood Pumps. Artificial Organs. 2013;38(4):316–26. doi: 10.1111/aor.12139 23889536 10.1111/aor.12139

[24] Yildiran T, Koc M, Bozkurt S, Sahin DY, Unal I, Acarturk E. Low Pulse Pressure as a Predictor of Death in Patients with Mild to Avanced Heart Failure. Texas Heart Institute Journal. 2010;37(3):284–90. 20548803 PMC2879196

[25] Jackson CE, Castagno D, Maggioni AP, Kober L, Squire LB, Swedberg K, et al Differing Prognostic Value of Pulse Pressure in Patients with Heart Failure with Reduced or Preserved Ejection Fraction: Results from the MAGGIC Individual Patient Meta-Analysis. European Heart Journal. 2015;36(18):1106–14. doi: 10.1093/eurheartj/ehu490 25616644 10.1093/eurheartj/ehu490

[26] Stergiopulos N, Westerhof N. Determinants of Pulse Pressure. Hypertension. 1998;32:556–9. 9740625 10.1161/01.hyp.32.3.556

[27] Castelain V, Herve P, Lecarpentier Y, Duroux P, Simonneau G, Chemla D. Pulmonary Artery Pulse Pressure and Wave Reflection in Chronic Pulmonary Thromboembolism and Primary Pulmonary Hypertension. Journal of American College of Cardiology. 2001;37(4):1085–92. 10.1016/s0735-1097(00)01212-2 11263613

[28] Ferguson JJ, Randall OS. Hemodynamic correlates of Arterial Compliance. Catheterization and Cardiovascular Diagnosis. 1986;12:376–80. 3815504 10.1002/ccd.1810120604

[29] Ramnarine KV, Nassiri DK, Hoskins PR, Lubbers J. Validation of a New Blood-Mimicking Fluid For Use In Doppler Flow Test Objects. Ultrosound in Medical & Biology. 1998;24(3):451–9. 10.1016/s0301-5629(97)00277-9 9587999

[30] Timms D, Gregory SD, Greatrex N, Pearcy M, Fraser J, Steinseifer U. A Compact Mock Circulation Loop For The In Vitro Testing of Cardiovascular Devices. Artificial Organs. 2011;35(4):384–91. doi: 10.1111/j.1525-1594.2010.01088.x 20883450 10.1111/j.1525-1594.2010.01088.x

[31] Gregory SD, Stevens MC, Timms D, Pearcy M, editors. Replication of the Frank-Starling Response in a Mock Circulation Loop. Annual Conference of the IEEE Engineering in Medicine and Biological Society; 2011 August 30—September 3, 2011; Boston, Massachusetts, USA. 10.1109/IEMBS.2011.6091683 22255906

[32] Klingensmith ME, Aziz A, Bharat A, Fox AC, Porembka M. The Washington Manual of Surgery. Philadelphia, USA: Lippincott Williams & Wilkins; 2011.

[33] Lankhaar JW, Westerhof N, Faes TJ, Gan CT, MK M. Pulmonary Vascular Resistance and Compliance Stay Inversely Related During Treatmet of Pulmonary Hypertension. European Heart Journal. 2008;29(13):1688–95. doi: 10.1093/eurheartj/ehn103 18349027 10.1093/eurheartj/ehn103

[34] Ghio S, Crimi G, Pica S, Temporelli PL, Boffini M, Rinaldi M, et al Persistent Abnormalities in Pulmonary Arterial Compliance After Heart Transplantation in Patients with Combined Post-Capillary and Pre-Capillary Pulmonary Hypertension. Plos One. 2017;1–10. 10.1371/journal.pone.0188383 PMC5703525 29176890

[35] Syyed R, Reeves JT, Welsh D, Raeside D, Johnson MK, Peacock AJ. The Relationship Between the Components of Pulmonary Artery Pressure Remains Constant Under All Conditions in Both Health and Disease. Chest. 2008;133(3):633–9. doi: 10.1378/chest.07-1367 17989160 10.1378/chest.07-1367

[36] Chemla D, Castelain V, Humbert M, Hebert J-L, Simonneau G, Lecarpentier Y, et al New Formula for Predicting Mean Pulmonary Artery Pressure Using Systole Pulmonary Artery Pressure. Chest. 2004;126(4):1313–7. doi: 10.1378/chest.126.4.1313 15486398 10.1378/chest.126.4.1313

[37] Grant BJB, Paradowski LJ, Fitzpatrick JM. Effect of Perivascular Electromagnetic Flow Probes on Pulmonary Hemodynamics. Journal of Applied Physiology. 1988;1988(65):4. 10.1152/jappl.1988.65.4.1885 3053589

[38] Cheng A, Williamitis CA, Slaughter MS. Comparison of Continuous-Flow and Pulsatile-Flow Left Ventricular Assist Devices: Is There An Advantage to Pulsatility? Annals of Cardiothoracic Surgery. 2014;3(6):573–81. doi: 10.3978/j.issn.2225-319X.2014.08.24 25512897 10.3978/j.issn.2225-319X.2014.08.24 PMC4250555

[39] Eda K. Optimal Pulse Pressure of Pulmonary Circulation under Bi-ventricular Assist After Cardiogenic Shock. Annals of Thoracic Cardiovascular Surgery. 1999;5(6):365–9. 10637385

[40] Noor MR, Ho CH, Parker KH, Simon AR, Banner NR, Bowles CT. Investigation of the Characteristics of HeartWare HVAD and Thoratec HeartMate II Under Steady and Pulsatile FLow Conditions. Artifical Organs. 2016;40(6):549–60. 10.1111/aor.12593 26611518

[41] Akimoto T, Yamazaki K, Litwak P, Litwak KN, Tagusari O, Mori T, et al Relationship of Blood Pressure and Pump Flow in an Implantable Centrifugal Blood Pump During Hypertension. ASAIO Journal. 2000;46(5):596–9. 11016515 10.1097/00002480-200009000-00018

[42] Sugiki H, Nakashima K, Vermes E, Loisance D, Kirsch M. Temporary Right Ventricular Support With Impella Recover RD Axial Flow Pump. Asian Cardiovascular Thoracic Annals. 2009;17(4):395–400. doi: 10.1177/0218492309338121 19713337 10.1177/0218492309338121

[43] Di Molfetta A, Iacobelli R, Filippelli S, Grutter G, Perri G, Iodice F, et al Evolution of Biventricular Loading Condition in Pediatric LVAD Patient: A Prospective and Observational Study. Artifical Organs. 2017 doi: 10.1111/aor.13050 29230826 10.1111/aor.13050

[44] Alfie J, Waisman GD, Galarza CR, Camera MI. Contribution of Stroke Volume to the Change in Pulse Pressure Pattern with Age. Hypertension. 1999;34:808–12. 10523365 10.1161/01.hyp.34.4.808

[45] Dart AM, Kingwell BA. Pulse Pressure-A Review of Mechanisms and Clinical Relevance. Journal of American College of Cardiology. 2001;37(4):975–84. 10.1016/s0735-1097(01)01108-1 11263624

[46] Milnor WR, Conti RC, Lewis KB, O'Rourke MD. Pulmonary Arterial Pulse Wave Velocity and Impedance in Man. Circulation Research. 1969;25:637–49. 5364641 10.1161/01.res.25.6.637

[47] Wang Z, Chesler NC. Pulmonary Vascular Wall Stiffness: An Important Contributor to the Increased Right Ventricular Afterload with Pulmonary Hypertension. Pulmonary circulation. 2011;1(2):212–23. doi: 10.4103/2045-8932.83453 22034607 10.4103/2045-8932.83453 PMC3198648

[48] Mitchell JH, Tardif J-C, Arnold JMO, Marchiori G, O'Brien TX, Dunlap ME, et al Pulsatile Hemodynamics in Congestive Heart Failure. Hypertension. 2001;28(6):1433–9. 10.1161/hy1201.098298 11751731

[49] Safar ME, Totomoukouo JJ, Asmar RA, Laurent SM. Increased Pulse Pressure in Patients With Arteriosclerosis Obliterans of the Lower Limbs. Arteriosclerosis. 1987;7(3):232–7. 3593069 10.1161/01.atv.7.3.232

[50] Millar JA, Lever AF. Implications of Pulse Pressure as a Predictor of Cardiac Risk in Patients with Hypertension. Hypertension. 2000;36(5):907–11. 11082165 10.1161/01.hyp.36.5.907

[51] Stenmark KR, Fagan KA, Frid MG. Hypoxia-Induced Pulmonary Vascular Remodeling. Circulation Research. 2006;99(7):675–91. doi: 10.1161/01.RES.0000243584.45145.3f 17008597 10.1161/01.RES.0000243584.45145.3f

[52] Thenappan T, Prins KW, Pritzker MR, Scandurra J, Volmers K, Weir EK. The Critical Role of Pulmonary Arterial Compliance in Pulmonary Hypertension. Annals of American Thoracic Society. 2016;13(2):276–84. 10.1513/AnnalsATS.201509-599FR PMC5461956 26848601

